# Self Multi-Head Attention-based Convolutional Neural Networks for fake news detection

**DOI:** 10.1371/journal.pone.0222713

**Published:** 2019-09-26

**Authors:** Yong Fang, Jian Gao, Cheng Huang, Hua Peng, Runpu Wu

**Affiliations:** 1 College of Cybersecurity Sichuan University, Chengdu, Sichuan, China; 2 College of Electronics and Information Engineering Sichuan University, Chengdu, Sichuan, China; 3 China Information Technology Security Evaluation Center, Beijing 100085, China; University of North Carolina at Chapel Hill, UNITED STATES

## Abstract

With the rapid development of the internet, social media has become an essential tool for getting information, and attracted a large number of people join the social media platforms because of its low cost, accessibility and amazing content. It greatly enriches our life. However, its rapid development and widespread also have provided an excellent convenience for the range of fake news, people are constantly exposed to fake news and suffer from it all the time. Fake news usually uses hyperbole to catch people’s eyes with dishonest intention. More importantly, it often misleads the reader and causes people to have wrong perceptions of society. It has the potential for negative impacts on society and individuals. Therefore, it is significative research on detecting fake news. In the paper, we built a model named SMHA-CNN (Self Multi-Head Attention-based Convolutional Neural Networks) that can judge the authenticity of news with high accuracy based only on content by using convolutional neural networks and self multi-head attention mechanism. In order to prove its validity, we conducted experiments on a public dataset and achieved a precision rate of 95.5% with a recall rate of 95.6% under the 5-fold cross-validation. Our experimental result indicates that the model is more effective at detecting fake news.

## Introduction

In recent years, social media has become ubiquitous and most important for social networking and content sharing [1]. Because of its instantaneity and convenience, people can quickly know what is happening recently and get a great quantity of information. The social media not only facilitates people’s life but also enrich people’s inner world. However, with the advantage of a broad audience and fast dissemination in social media, the fake news can quickly go viral as well.

In the age of information explosion, without analyzing and judging the massive information effectively, the information will become a burden. The fake news is deceptive, always misleading people, what’s worse, it is difficult to distinguish. A study reported that many people typically retweets false information on Twitter [2]. What’s worse, falsehood diffuses significantly farther, more quickly, deeper, and more broadly than the truth in all categories of information [3]. When more and more people accept the fake news and take its content as real-life events, it may have a significant impact on the fields of politics, finance, military science, education and cause great damage to the society. For examples, after the 2016 election, speculation about the influence on the election outcome dominated political discourse [4], it easily leads to upheaval. And the $130 billion in stock value are wiped out quickly because the fake news was claiming that Barack Obama was injured in an explosion [5].

In this new world of social media, with the increase of network traffic, it still is challenging for fake news detection. Firstly, the content of fake news become more diverse in terms of topics, styles and media platforms [6]. It is difficult to extract obvious features from these rich and colourful contents. Secondly, to invert black and white and mix truth and fiction intentionally, some real events will be incorporated into the fake news. For example, fake news may cite realistic evidence, data to support an inauthentic point or conclusion in the context. It generally increases the difficulty of extracting the manual feature based on the news content.

Deep learning methods have achieved tremendous success in the field of pattern recognition. In the problem of classification, such as text classification and images classification, it also has shown the efficiency and be used extensively. The attention mechanism was initially used in the field of image processing, and in recent years, it has been gradually used in the field of NLP (natural language processing) because of its peculiarity. Now, it is trend using it to get a more accurate classifier in the deep learning frameworks. The attention mechanism can get the relation between the whole and the part in one step and help the classifier grasp the easily overlooked but significant features from the raw data.

In this work, we studied a fake news model that takes advantage of CNN (convolutional neural networks) and self multi-head attention mechanism. To ensure its accuracy, we applied it to a public dataset and compare with several popular baseline methods. We also demonstrated its generalization capabilities by evaluating a topic that is not included in the training dataset. To obtain a more objective evaluation of results, we compared the accuracy of our fake news classifier with previous experimental results. The result showed that higher accuracy can be acquired in fake news detection with this proposed classifier. Furthermore, we also backtracked the output of classifier and got the pivotal word that makes more effect on the processing of detection. The paper’ contributions can be summarized as follows:

- We built a model to detect the fake news by combining the advantages of the convolutional neural networks and the self multi-head attention mechanism.
- The proposed model got quality results in fake news detection, and achieved an accuracy rate of 95.5% under 5-fold cross-validation in the public dataset. Also, by comparing the accuracy with previous work, and the result showed that the proposed classifier can get more outstanding performance.
- We backtracked the output of classifier, and highlight the keywords in the news that contribute to the classification.

The rest of the paper is organized as follows. Section 2, we have a review of related work about fake news detection and the attention mechanism. Section 3, we introduce the proposed model architecture in detail. Section 4, we present the dataset used in this work, and we show the experimental environment, the experimental evaluation standard, and experimental operation. Next, we show the experimental results and evaluate it with previous work. Finally, we select the words that contribute to the classification by backtracking the classification result. Section 5, we summarize conclusion and propose future works.

## Related work

Fake information detection is a hot topic in the past few years. In the meantime, the plentiful and complex of fake news has enriched the form of misinformation. Sometimes, rumor and misinformation are also regarded as fake news. But in fact, news usually reports a complete story. That indicates detect the fake news does not conform to other fake information, such as fake short comments and fake short title. Therefore, in this study, we define the fake news be detected is news reports or articles that its content exists bogus and unveracious information. At that point, we summarize the previous relevant research according to two perspectives: based on the news content and based on the news context.

### Content-based in fake news detection

From different angles, the features that were extracted are different based on the content of the news. Generally speaking, the content of the news includes the headline and the body text. The headline is a short title that summarizes the topic of the news article and the detailed information is presented in the body text. Based on these raw content attributes, the characteristics of fake news can be extracted. Such as extracting different text features of statements or headlines to detect fake news by using traditional machine learning algorithms and NLP-techniques [7]. Moreover, the techniques of word embedding and deep neural networks have attracted much attention to textual feature extraction, especially in the problem of classification, which has shown good performance. Some researchers have investigated in the field of detection the fake news, the fake information, and achieved quality results [8–12].

In addition to analyzing the text, the writing style of news also can be captured. The author of news often has their writing characteristics which are reflected in the habit of using syntax and rhetorical structure, some researchers have investigated it. Feng S et.al. [13] researched syntactic stylometry for deception detection and Afroz S et.al. [14] proposed a method for detecting stylistic deception in written document. Potthast M et.al. [15] demonstrated a new way of assessing style similarity text categories via unmasking—a meta-learning. The detection method based on the writing style often can extract deep, inconspicuous features from news content, even can tell whether the author of news is a robot [16].

The content of the news article includes not only text information, but also a large number of detailed auxiliary images. It also conveys some valuable information. Visual cues are an essential manipulator for fake news propaganda. Fake news producers often use fake images or videos to stimulate the emotions of readers, and may some major misinformation is just expressed in it. From these visual elements, different characteristics of fake news can also be captured. Gupta A et.al. [17] got a high accuracy in predicting fake images from real images of Hurricane Sandy by using Decision Tree Classifier. Jin Z et.al. [18] proposed several visual and statistical features to identify these patterns visually and statistically for detecting fake news. They all achieved acceptable results. Analyzing non-text elements such as pictures and videos that will increase the burden of model training but simultaneously expand the dimension of the feature, which is helpful to improve the accuracy of the detection model.

### Context-based in fake news detection

The main information that the news aim to convey is present in the content of news articles, it is visible and not hard to get for us. Rather, the report context is not visualized, people may ignore it, but it also plays an essential role as additional information in the news article. The news context usually includes the author, published time, page views, degree of popularity, transmission rates, comment on the news article, and so on. Through analyzing the origination and the circumstance of the news article that spread on social networks, the authenticity of it can be inferred. Each user has the fingerprint to represent its unique, in the social network, the profile, content, and others constitute its unique features. By aggregating author’s flags, it is possible to determine the authenticity of the article. Tschiatschek S et al. [19] considered the leveraging crowd signals to detect fake news. Rumours find no credence with a wise man. Some researcher also takes advantage of the reader’s feedback to improve news classification accuracy by mining conflicting viewpoints in the news article [20–22].

Cross-linking tends to produce excellent results. Ruchansky N et.al. [23] tried to create a model with an automated prediction and more accurate by combining three general characteristics: the text of news article, the content of news, the user responses and the source writer. That is pretty complex work, and in the actual detection processing, it needs detailed raw news as much as possible.

### The attention mechanism

The attention mechanism was initially applied in the field of image processing, it can effectively capture images’ local features. Just like human vision, the partial can generalize the whole, and people may pay more attention to certain parts of the picture. Considering its characteristics, Bahdanau D et al. [24] took it into the machine translation task and got a high-quality result. Then the attention mechanism is widely used in the field of NLP and combines with LSTM (short-term long memory), convolutional neural networks and others to solve some classification problems. Such as classification of sentences [25, 26], relation classification [27, 28] and the detection of satirical news [29, 30].

News has the characteristics of real-time, thus it is necessary to detect fake news as soon as possible. We have summarized two methods for detecting fake news: context-based in fake news detection and content-based in fake news detection. For the context-based in fake news detection, not every news has sufficient contextual information, also, in the process of actual detection, to collect the contextual information is not easy and will take some time, then the fake news may have been spread and caused some impact on some filed. Therefore, the context-based fake news detection method is not suitable for timely detection. For the content-based in fake news detection, news content is the information what we can get rapidly. Compared with the context-based detection method, the content-based fake news detection is more real-time and can judge the authenticity of news more quickly in the process of actual detection. In previous work, the content-based fake news detection methods often use CNN, LSTM, and other deep learning algorithms to extract news content features for detection. Although the detection results are significant, they are not intuitive enough and have some defects due to the characteristics of the algorithm. For example, the basic CNN architecture can get the local information by the convolution kernels, but because of the characteristics of stride and the size of convolution kernels, it cannot capture the contextual information and the correlations between the widely separated words, it will affect the detection performance of the model. Then cannot objectively explain how the model detects fake news. Hence, we learn to detect fake news based only on its contents and use the attention mechanism in the model to improve the accuracy of detection, which can mitigate the problem effectively.

## The proposed model architecture

To detect fake news based only on its contents, we proposed an approach for building a hierarchical neural network model architecture with convolutional neural networks and self multi-head attention mechanism. The self multi-head attention mechanism is an expert at obtaining the internal spatial relationship in words. Then the attention mechanism combined with CNN can make up for the lack of basic convolutional neural networks that cannot obtain the connections between distant words effectively.

**Fig 1:**
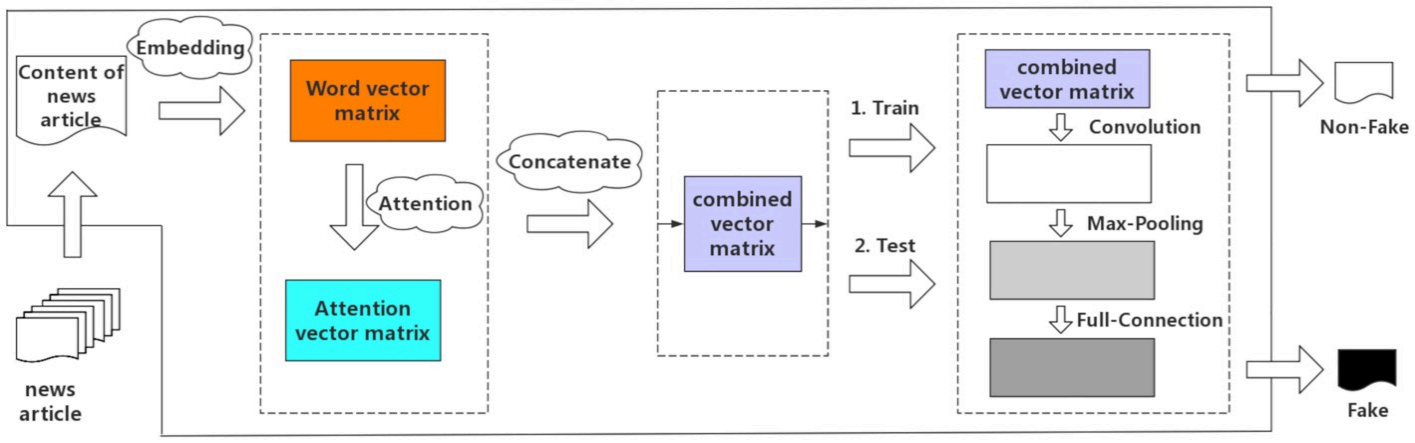
The architecture of the SMHA-CNN model.

As shown in Fig 1, not like a basic normal convolutional neural networks architecture [31], we introduce the attention mechanism rather than a convolutional layer after the layer of word embedding.

### Word embedding

The word embedding is widely used in natural language processing. It maps the words or phrases to a real value vector which can reflect the semantic distance, the relationship between words. In this architecture, we use public pre-trained word vector model to accomplish word embedding. As the Eq 1 shows. The word vector model replaces each word with its corresponding vector and creates a news article matrix $X\in R^{n\times d}$ for next operation. Where the *n* is the number of the word in the article, and the *d* is the dimension of word embedding,

$$X=(x_{1},x_{2},\ldots ,x_{i})$$ (1)

where $x_{i}\in R^{d}$ is the *d*-dimensional word vector corresponding to the *i*-th word in the news.

### Self multi-head attention mechanism

The word vector is the feature representation of the word. And the word matrix that can well reflect the meaning and internal relationship of words is the premise of obtaining a high accuracy classification model. However, the basic word vector model only reflects the semantics of individual words rather than the relationship between words. The self multi-head attention mechanism [32] is an expert at obtaining the internal spatial relationship in words, which can be used to mitigate the problem.

The self multi-head attention mechanism is the improvement of the basic attention mechanism, and the basic attention mechanism is defined as the following equation.

$$Attention\left(Q,K,V\right)=\text{softmax}(\frac{QK^{⊤}}{\sqrt{d_{k}}})V$$ (2)

where $Q\in R^{n\times d_{k}}$,$K\in R^{m\times d_{k}}$, $V\in R^{m\times d_{v}}$. *Q* inner product with *K*^⊤^, and the $\sqrt{d_{k}}$ adjust its value, then use the activation function of *softmax* to product a new value inner product with the *V*, and product a new vector. The new vector reflects the similarity between *Q* with *V*.

Multi-head attention mechanism collects the different levels of information from raw data, the equations are describing as the following:

$$head_{i}=Attention(QW_{i}^{Q},KW_{i}^{K},VW_{i}^{V})$$ (3)

$$Multi-Head\left(Q,K,V\right)=Concat(head_{1},\ldots ,head_{h})$$ (4)

SelfMulti-Head=Multi-Head(X,X,X) (5)

These three equations are the formation process of the self multi-head attention mechanism. The matrix of *W* is the weight matrix. The *Q*, *V*, *K* should multiply its corresponding weight matrix before getting into the attention function. Repeat this process *h* times and connect each result then we can get a new vector matrix that reflects the relationship between *Q*, *V*. Especially, in the self multi-head attention mechanism, to look for internal connections within words, the *Q* = *V* = *K* = *X*, and the *X* represents the word vector matrix.

The multi-head attention mechanism helps the model learn the words relevant information in different presentation subspaces. The self-attention mechanism can extract the dependence in words. As the name shows, the self multi-head attention mechanism integrates the benefits of both, creates a context vector for each word. Then we don’t need to depend on additional information and get a matrix that reflects the abundant context relationship between each word and others.

### Concatenate word vector matrix and attention matrix

If each word vector cannot adequately express the meaning of each word in the news, it will be a big challenge to extract feature. Because the characteristics of the convolutional neural network cannot get the correlation between non-consecutive words, we need a unique word vector matrix that not only can represent the semantics of a word but also can reflect the contextual information between words.

We can get an attention matrix that reflects abundant context information according to the self multi-head attention mechanism, and concatenated it with the basic news article’s word vector matrix, then got a new extended news article matrix. The collective matrix enriches word vector of each word, particularly makes up for the word embedding of Word2vec and other word embedding models that cannot capture contextual information between non-consecutive words. That rich word vector matrix helps the next processing pay more attention to the specific significant words and extract more obvious classification features during the process of model training. The following equation shows the details of the new matrix formation.

$$Att_{matrix}=({Att}_{1},{Att}_{2},\ldots ,{Att}_{i})$$ (6)

$$New-matrix=Concat(Att_{matrix},Art_{matrix})$$ (7)

where *Att*_*i*_ ∈ *R*^*d*^ is the *d*-dimensional attention word vector corresponding to the *i*-th word in the news, It can reflect the context information of the *i*-th word. Where the *Art*_*matrix*_ is the news article’s word vector matrix that is built from the word vector model, it cannot reflect the internal relationship of the words.

The *Art*_*matrix*_ and *Att*_*matrix*_ are both two-dimensional matrices, spliced them on the Y-axis and build a large matrix. Then each row of the collective matrix represents the corresponding word adequately, help the following process to extract more effective features.

### Convolution, polling and fully connected layer

The convolution, pooling, full connection layers are the standard layer in the basic convolutional neural network framework. Because only the entire word vector that from the concatenate layer can fully represent the word information, we use the 1D-convolutional mechanism to deal with the matrix, then the more accurate and more effective features will be extracted by the convolution kernels. In the layer of polling, we use the max-pooling mechanism to reduce the impact of noise that from the output of convolutional layer, at the same time, use it can reduce the feature dimension and prevent model overfitting. Finally, using the fully connected layer to connect every extracted feature and using the activation function of *softmax* to classify the news.

## Experiments

### The data and experimental environment

The datasets used in the paper are collected from the **fakenews.mit.edu**, and it has already provided a full introduction to the dataset. The public fake news dataset with approximately 24,000 news articles published between October 26 and November 25, 2016. Consisted of approximately 12,000 articles pulled from Kaggle, which maintains a blacklist of fake news source websites and the real news dataset was comprised of 9,000 Guardian articles and over 2,000 New York Times articles. We used 12,228 fake news and 9,762 non-fake news in the experiment finally.

The experimental environment is shown as Table 1. Based on the advantages of simple, practical and using habit, we used Keras to construct the proposed classifier.

**Table 1:** Experimental environment configuration.

Items	Configuration
OS	Ubuntu 16.04.3 LTS
The system configuration	CPU:Intel i7-7700,RAM:16G
	GPU:GeForce GTX 2080 8G
The library of Python	Keras,Scikit-learn,Matplotlib

### Metric

Because this classification task is a binary decision problem, the detection result of news may either be fake or non-fake.

As shown in Table 2, the confusion matrix has four categories: TP indicates that predicting the fake article samples are fake. FP indicates that predicting the non-fake article samples are fake. In the same way, FN indicates that predicting the fake article samples are non-fake. TN indicates that predicting the non-fake article samples are non-fake. We use precision, recall, f1-score and accuracy to measure the performance of the classifier. The specific calculation formula is shown as follows:

$$Precision=\frac{TP}{TP+FP}$$ (8)

$$Recall=\frac{TP}{TP+FN}$$ (9)

$$F1-score=\frac{2*Precision*Recall}{Precision+Recall}$$ (10)

$$Accuracy=\frac{TP+TN}{TP+TN+FP+FN}$$ (11)

**Table 2:** Confusion matrix.

	Actual fake	Actual Non-fake
**Predicted fake**	TP	FP
**Predicted Non-fake**	FN	TN

As shown in equations, the precision rate represents the accuracy of predicting positive samples, and the recall rate represents the probability of being predicted positive samples in the actual positive samples. The accuracy rate can judge the accuracy rate of the model, and the f1-score comprehensively reflects the precision rate and accuracy rate. The higher the values, the better the prediction result of the model. Also, the receiver operating characteristic curve (ROC) can intuitively reflect the performance of the model. The larger the area under the curve (AUC), the better the classification performance of the model. These five measurement indexes are used to verify the validity and practicability of the model.

### Training process

#### Clean data

**Fig 2:**
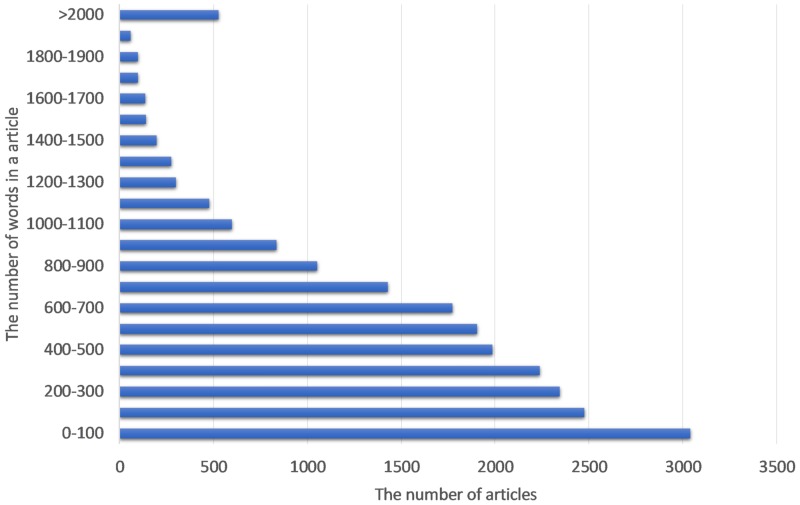
The distribution of raw article length.

The training data was the raw news articles, in those news articles, there was a great quantity of information that is useless to train the model, and we need to clean it. Firstly, remove the punctuation in the articles, then used the common word segmentation method to deal with the articles. Secondly, remove the common but insignificant words and expressions in the news articles, such as ‘read more’, ‘Share on Facebook’. After cleaning the article, counter the number of words in each article. As shown in Fig 2, Most articles are within 1,200 words, so we took out the first 1,200 words of each article as the model’s input.

#### Word embedding

For comparison, we used two different pre-trained word vector models for word representation. It is Golve and Word2vec-GoogleNews-vectors-negative300. The two models use different mechanisms to replace each word with its corresponding pre-trained vector. They both are classical word vector model and used widely in the task of needing word embedding.

#### Model setting

In the model training, ninety percent of the data was for training model, and ten percent of the data was for validating model practicability and validity. The 1D-convolutional layer with 128 filters of size equal to three words. The dropout_ratio was 0.5, and the function of *ReduceLROnPlateau* was used to monitor the loss of validation as the optimizer.

#### Data feed

We conducted two groups of experiments. In the experiment *A*, we evaluated the performance of the classifier under 5-fold cross-validation. The proportion of the positive and negative samples in each of the five subsets is the same as the overall data, and each subset used exactly once as validation data. For objective evaluation, we also compared our model with several competitive baseline methods. At the same time, we also did experiment *B* by using the same data feed in [8], it only uses the CNN method to detect fake news, so the SMHA-CNN method can be well contrasted with it. In this control experiment, we also held out the topic named “Trump”. The news articles containing “trump” words had not participated in the training process of the classifier model and only used these articles to evaluate the model. It was objective to validate the accuracy and reliability of the classifier.

### Evaluation and result

#### Result in experiment A

In this experiment, the final result is shown as Table 3. The method of SMHA-CNN-1200’s precision reached 95.5% in the detection of news articles with Word2vec. Meanwhile, on the samples with different labels, the rate of precision and recall are also high. Comparing our model with several competitive baseline methods of LSTM [33], Att-LSTM [34] (use attention mechanism in LSTM), Bi-LSTM, GRU [35]. LSTM with text information is inefficient with very long sequences, and the model with 1200 as input length performs very worse. Hence, we also take the 400 as its input length. For objective comparison, we did the same thing on the other methods. SMHA-CNN outperforms all the baseline methods significantly.

**Table 3:** The result of using 5-fold cross validation with Word2vec in experiment A.

Method		Precision(%)	Recall(%)	F1-score(%)
**SMHA-CNN-1200**	non-fake	94.5	95.6	95.1
	fake	96.5	95.6	96.3
	avg/total	**95.5**	**95.6**	**95.6**
**LSTM-1200**	avg/total	67.3	67.5	67.1
**Att-LSTM-1200**	avg/total	91.1	91.0	91.0
**Bi-LSTM-1200**	avg/total	86.2	86.1	86.1
**GRU-1200**	avg/total	82.3	81.2	80.7
**SMHA-CNN-400**	avg/total	**93.8**	**93.8**	**93.8**
**LSTM-400**	avg/total	83.0	83.0	83.0
**Att-LSTM-400**	avg/total	88.6	88.5	88.5
**Bi-LSTM-400**	avg/total	85.5	85.5	85.5
**GRU-400**	avg/total	92.6	92.6	92.6

**Fig 3:**
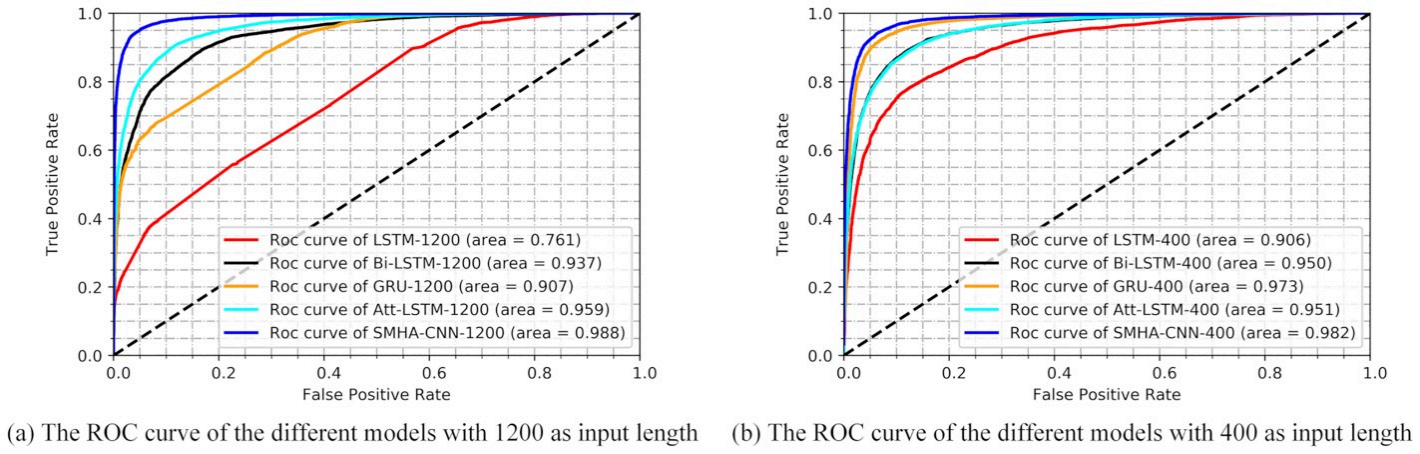
The receiver operating characteristic curve of the different models. (a) Using the number of 1200 as input length, the red curves show the performance of LSTM models, the black curves show the performance of Bi-LSTM model, the orange curves show the performance of GRU model, the green curves show the performance of Att-LSTM model, the blue curves show the performance of SMHA-CNN model. (b) Using the number of 400 as input length, and the red curves show the performance of LSTM model, the black curves show the performance of Bi-LSTM model, the orange curves show the performance of GRU model, the green curves show the performance of Att-LSTM model, the blue curves show the performance of SMHA-CNN model.

Try to get a visualized result, a receiver operating characteristic (ROC) curve is plotted as shown in Fig 3. We earned a perfect ROC curve with the area under curve (AUC) value of 0.988.

#### Result in experiment B

The result is shown as Table 4. In this work, the model shows better performance than the result in [8] that only use CNN to detect the fake news. When using all the article in the experiment with Word2vec. The proposed classifier’s precision is up to 95.9%, and the accuracy rate is up to 95.90%, which is higher than the result of 93.5% in [8]. The more intuitive result produce in the experiment of hold-out the topic named “Trump”. The accuracy rate is up to 93.11%. It is obviously higher than the result of 87.7%, and up 5.4 percent. These data fully demonstrate that using the self multi-head attention mechanism in the convolutional neural networks can indeed improve the accuracy of detecting fake news.

**Table 4:** The evaluating results in experiment B.

	precision(%)	recall(%)	f1-score(%)	accuracy(%)
**All news (Word2vec+SMHA-CNN)**	95.9	95.9	95.9	**95.90**
**All news (Glove+SMHA-CNN)**	94.7	94.7	94.7	94.67
**All news in** [8]**(Word2vec+CNN)**	-	-	-	93.5
**Hold out “Trump” (Word2vec+SMHA-CNN)**	93.1	93.1	93.0	**93.11**
**Hold out “Trump” (Glove+SMHA-CNN)**	91.5	91.2	91.0	91.19
**Hold out “Trump” in** [8]**(Word2vec+CNN)**	-	-	-	87.7

#### Visualization

Visualization is informative that can reflect what be focused on the network layer. By backtracking the output of the dense layer, we get the dense layer’s weights and multiply it by the max-pooling layer units. We select top-10 units with the largest values from the result, so these top-10 units contribute the most to the classification. After that, we react it to the max-pooling layer by the indexed again. We use one-dimensional convolution technique in convolution layer, so we can trace the units back to the convolutional layer and mark the actual words easily. Also, because the one-dimensional convolution with 128 filters of size equal to 3, the one unit will correspond to three words, so that different units may be mapping to the same words.

**Fig 4:**
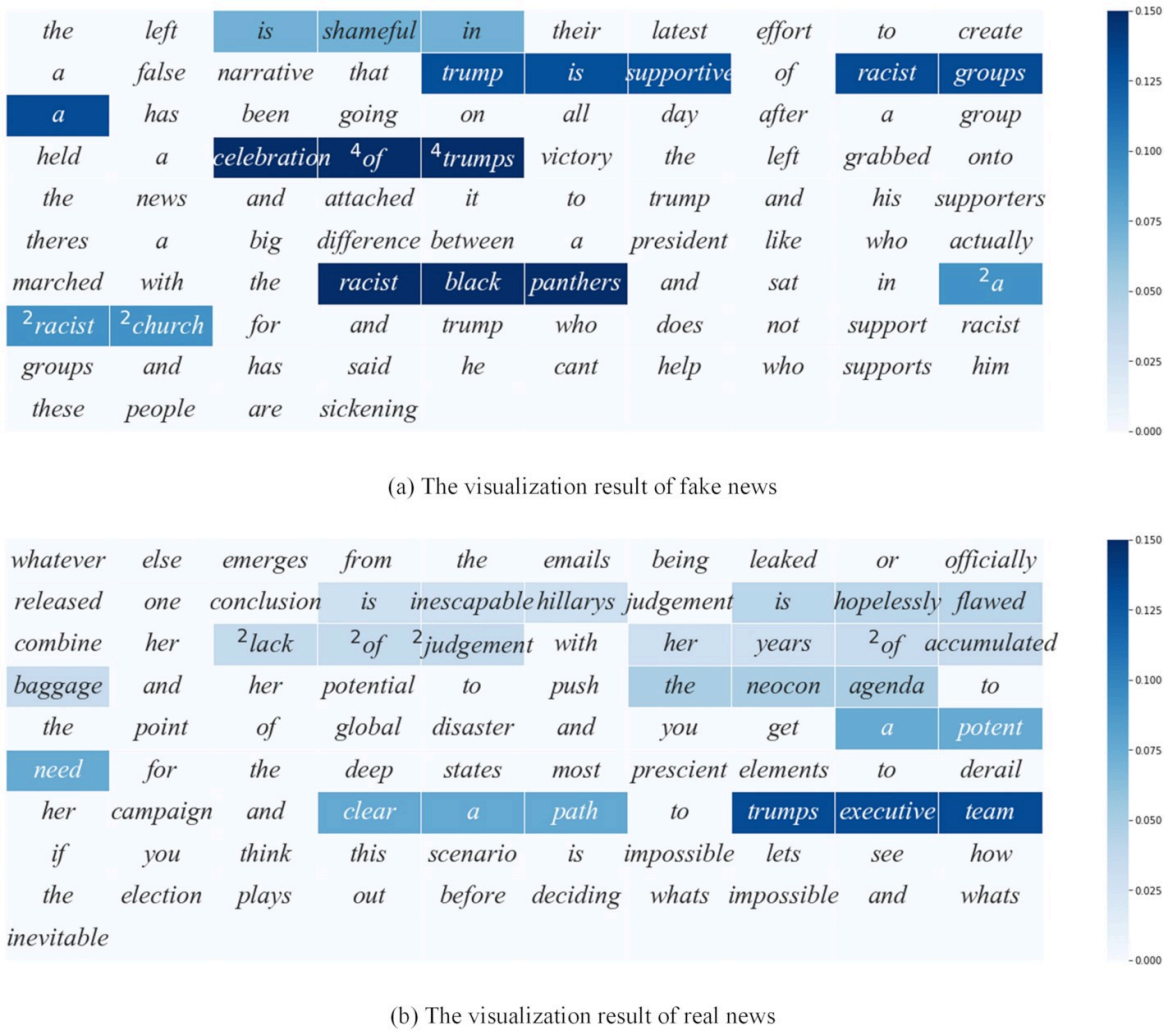
The different samples’ visualization results. (a) The visualization result of a sample of fake news, the number in the top left corner of the word represents the times that the word be mapped. (b) The visualization result of a sample of real news, the number in the top left corner of the word represents the times that the word be mapped.

Fig 4 respectively shows the visualization of a fake news sample and a real news sample from the dataset. The words that are most useful for the classifier are highlighted, and the darker of a cell and the more contribution to the classifier.

As shown in Fig 4(a), the word of *racist* is highlighted several times and the phrase of *celebration of trumps* repeats in four units. It means that they are beneficial for classification in this single article. Also, as shown in Fig 4(b), more adjectives with emotional overtones are highlighted. such as *inescapable*, *hopelessly*, *accumulated*, *potent*. The visual displays are intuitive to show the core concerns of the classifier model, and it helps people understand what the language pattern be captured in the process of detecting fake news clearly.

## Conclusions and future work

To purify the environment of the Internet, the detection of fake information is urgent. In this paper, we proposed an effective model to detect fake news only based news content by using self multi-head attention-based convolutional neural networks. On detecting a novel topic, the proposed model still had excellent performance. Also, from the different and discriminating accuracy of the classifier in all topics, hold out the topic, and different word vector model, we can see that it is really important to measure the generalization capability of the classifier and it is equally essential in the layer of word embedding to choose the appropriate word vector model. Besides, we compared the experimental result with previous work objectively, and the result had shown that our proposed model of using self multi-head mechanism with the convolutional neural networks indeed can get more outstanding performance. In addition, we selected the most conducive words to classification by tracing the output of the dense layer in our proposed model architecture and give a visual display, to a certain extent, which can explain what the model cares about when detecting fake news.

In future work, another possible research direction is to understand how the classifier detects the fake news deeply, even how to modify or replace some keywords with similar meaning words to avoid the detection method that based on the semantics of fake news.
